# Evaluation of preclinical efficacy of human umbilical cord mesenchymal stem cells in ankylosing spondylitis

**DOI:** 10.3389/fimmu.2023.1153927

**Published:** 2023-03-30

**Authors:** Danpeng Shen, Zhiqiang Wang, Hongwei Wang, Hongyan Zhu, Cuibao Jiang, Fan Xie, Hongpeng Zhang, Qian Lv, Qi Liu, Nianmin Qi, Hao Wang

**Affiliations:** R&D Department, Asia Stem Cell Regenerative Pharmaceutical Co. Ltd, Shanghai, China

**Keywords:** hUCMSCs, ankylosing spondylitis, PGISp, efficacy evaluation, inflammatory cytokines

## Abstract

**Objective:**

Umbilical cord mesenchymal stem cells (UCMSCs) have significant regenerative, tissue repair, and immunomodulatory properties that can help reduce inflammatory responses in patients with ankylosing spondylitis (AS). In this study, we used a combination of bovine proteoglycan and dimethyldioctadecylammonium (DDA) to establish a mouse model of proteoglycan-induced spondylitis (PGISp). To evaluate the therapeutic effects of UCMSCs, we treated PGISp mice with different doses of hUCMSCs *via* tail vein injection.

**Methods:**

At week 13, the PGISp mice exhibited thickened, erythematous paws, erythema in the extremities, and lameness. CT scans revealed necrotic lysis of chondrocytes, formation of fissures, visible hemorrhage, connective tissue hyperplasia, and focal infiltration of lymphocytes in the intervertebral discs. At week 14, the PGISp mice were randomly divided into three groups and administered different doses of hUCMSCs (0.25, 0.5, and 1.0×10^7^ cells/kg, iv, QOW×2, n=10). To assess the therapeutic effects of hUCMSCs, we evaluated Th cell subsets in the spleen, spleen and thymus coefficients, peripheral blood inflammatory factors, and pathological and imaging observations of the spines and lumbar spines in the PGISp mice.

**Results:**

The results demonstrated that injection of hUCMSCs shifted the balance axis between Th1 and Th2 cells in the spleen towards Th2 cells. Moreover, the spleen coefficient and levels of inflammatory cytokines (TNF-α and CCL-2) in the serum decreased after hUCMSC injection. CT imaging and pathological analysis indicated that hUCMSC treatment inhibited ectopic osteogenesis and maintained clear small joint gaps, which slowed down the progression of structural lesions in the disc, nucleus pulposus, fibrous ring, and cartilage in PGISp mice.

**Conclusion:**

Administering hUCMSCs at the 14th week after modeling proved to be an effective treatment for PGISp mice. This experiment offers a valuable reference for the pre-clinical use of hUCMSCs in the treatment of AS.

## Introduction

Ankylosing spondylitis (AS), a form of spondyloarthritis, is an autoimmune disease that primarily involves the spinal joints, sacroiliac joints, and their adjacent soft tissues, such as tendons and ligaments. Retrospective epidemiological studies have shown that the prevalence of AS in the United States ranges from 0.2% to 0.55% (1). The average prevalence of AS in mainland China is 0.29% and continues to increase with the majority in men being 2.8 times higher than in women (2). AS expresses various clinical signs and symptoms, foremost among these are the most common being chronic back pain and progressive spinal stiffness. AS is also associated with multiple joint complications such as spinal and sacroiliac joint, peripheral joint, finger, and attachment point involvement. The disease progresses to the middle and late stage performs severe symptoms such as impaired spinal mobility, abnormal posture, hip pain, peripheral arthritis, attachment point inflammation, and finger inflammation (sausage finger), which seriously affects the patient’s normal life (3). The most common extra-articular manifestations of AS include inflammatory bowel disease (up to 50%), acute anterior uveitis (25% to 35%), and psoriasis (about 10%) (4). Studies have shown that AS also increases the incidence of cardiovascular disease, pulmonary complications, and vertebral fragility fractures due to systemic inflammation and decreased spinal mobility (5, 6).

Currently, effective treatments for AS are limited and are divided into traditional, pharmacological, and surgical treatments (7, 8). The treatment aim is mainly to relieve the patient’s signs and symptoms, restore physical function, prevent joint damage, improve the patient’s quality of life, and prevent complications of spinal disorders. Traditional treatments commonly used for AS including physical therapy and exercise, but they do not involve of solving the root cause of AS and thereby having very limited effects on relieving symptoms (9). Clinically, non-steroidal anti-inflammatory drugs (NSAIDs) and tumor necrosis factor alpha (TNF-α) inhibitors are the mainstays of treatment for AS, other pharmacological treatments include salazosulfapyridine, glucocorticoids, IL-6 receptor inhibitors (Sarilumab), IL-17 receptor inhibitors (Cosentyx), IL-12/23 receptor inhibitors (Ustekinumab), and Wnt signaling pathway inhibitors (10–12). However, continuous use of NSAIDs has not shown clinical benefit and may lead to the development of peptic ulcers, depression, and hypertension and increase the risk of cardiovascular disease (13–16). The use of receptor inhibitors such as TNF-α inhibitors, IL-6 and IL-17A is limited. One of the reasons is these inhibitors cannot be used in patients with active infections, tuberculosis, advanced heart failure, lupus, multiple sclerosis, and cancer, the other is these inhibitors produce a no response in 40% of patients, and thus requiring prompt adjustment to other treatment regimens (17, 18). In some patients with severe disease, surgery may be required if pain or extreme swelling occurs rapidly. None of the above treatments are currently able to slow the progression of AS and promote recovery from structural damage to the spines and other joints, making it difficult to address the root cause of the patient’s condition. Additionally, the treatment of AS is currently unable to slow the progression of the disease or promote recovery from structural damage to the spines and other joints (19–21).

In summary, AS requires more aggressive and effective modalities or means of treatment which can effectively suppress the systemic inflammatory response during all stages of AS disease progression, promote spinal repair during all stages of the disease, improve and maintain spinal flexibility and normal posture, relieve symptoms, reduce functional limitations, and reduce other complications such as peripheral joint development (22). Numerous studies suggest that stem cells have immunomodulatory, tissue repair, and regenerative effects and may have a greater potential as a therapeutic tool for the effective treatment of AS (23–25). Meanwhile, a series of clinical studies have been conducted to report the use of stem cells in patients with AS, and good results have been demonstrated on the safety and efficacy of hUCMSC (8, 26). The results of clinical studies have shown that hUCMSCs are safe and well tolerated in the treatment of AS patients, with a significant decrease in bath ankylosing spondylitis disease activity index (BASDAI) and bath ankylosing spondylitis functional index (BASFI) scores (27), significant improvement in blood sedimentation, C-reactive protein (CRP), Intercellular adhesion molecule (ICAM), and TNF-α levels, and an improvement in imaging scores (8, 28–31).

To further elucidate the underlying pathological mechanisms for AS, researchers established a series of tonic animal models including transgenic animal models, inflammation-induced animal models, and other animal models, according to different pathogenic mechanisms (32). Based on the strong correlation between AS and Human leukocyte antigen (HLA)-B27, several lines of HLA-B27 transgenic rat models have been developed in Lewis or Fisher rats by transfecting human HLA-B*2705 heavy chain and the invariant chain β2-microglobulin (hβ2m), which stabilizes the confirmation of major histocompatibility complex (MHC), the clinical manifestations of this model are very similar to AS, but the symptoms are mild, the modeling cycle is long, the operation is cumbersome, and the modeling rate is extremely low (33). The SKG mouse model carries a point mutation in the SH2 domain of Zeta-chain associated protein kinase 70 (ZAP-70). The diseased parts of the model are mainly concentrated in the limbs and joints, and the influence on the spines of the model mice is low (34). Inflammation-induced AS models mainly include animal models induced by proteoglycan, TNF-α, and LPS. The disease progression of PGISp models is very similar to the clinical manifestations of AS (35, 36). Then it develops upward along the spines, invading multiple intervertebral discs, and the operation is convenient, concise, short in cycle, and high in molding rate, but it shows very little genetic correlation. Proteoglycans are derived from cartilage, and cartilage is not monitored by the immune system. When cartilage is damaged or degraded by external factors or proteases, specific antigenic epitopes will be exposed, T cell recruitment will be induced, and cellular immune responses will occur, resulting in inflammatory damage similar to AS (37). The molding rate of animal models induced by TNF-α and LPS is low, and it can only simulate some symptoms of AS, such as enteritis, peripheral arthritis, etc.; Others, such as DAB arthritis model and progressive sclerosis model, mainly involve peripheral joints and joints, with minimal impact on the spines, and the clinical symptoms are not typical (32). Therefore, we used hUCMSCs to treat the PGISp mice to observe whether hUCMSCs could alleviate AS-related symptoms and to explore the quantitative efficacy relationship between the two within a certain range of doses.

## Methods

### Experimental design

Eighty SPF-grade female BALB/c mice, weighing approximately 17-19 g, were purchased from Zhejiang Viton Lever Laboratory Animal Technology Co. The mice were housed and used with the approval of the ethics committee of the Shanghai Institute of Pharmaceutical Industry and in compliance with all applicable institutional and governmental regulations regarding the ethical use of animals. After acclimatization, mice were injected intraperitoneally with a mixture of 1 mg/ml proteoglycan solution and 10 mg/ml DDA micelles at 0.2 ml per mouse at 3-week intervals according to 1:1 (v:v), i.e., the second and third sensitization reactions were performed at weeks 3 and 6, respectively (33).

After the third immunization, the extent of lesions on the extremities of mice was observed twice a week, and the mice in each group were selected for CT photography in week 13. In week 14, all mice were stratified and randomly grouped to ensure the number of animals in each group was 10, and thereafter therapeutic administration was started, intravenously, at one-week intervals, twice. The low, middle and high doses of treatment groups were designed as 2.5×10^6^, 5×10^6^, 10×10^6^ cells/kg, respectively, and the same dose of PBS was used as placebo in the control group.

**Figure 1:**
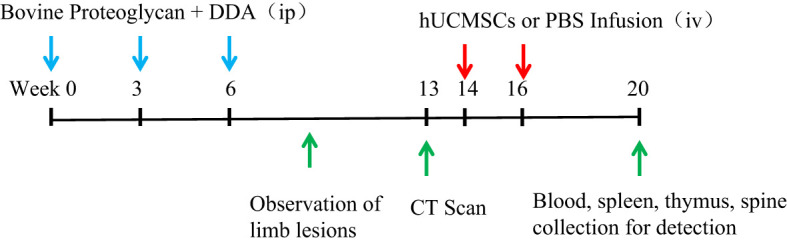
Schematic diagram of hUCMSCs treatment of AS. The blue arrows indicate the time points of administration of modeling reagents; The red arrows indicate the time points of administration of hUCMSCs or PBS; The green arrows indicate the time points of observation and detection.

At the 20th week, the blood of the mice was collected to detect the content of inflammatory factors and chemokines, the spleen and thymus were collected to weigh and calculate the organ coefficient, and the proportion of T cell subsets in the spleen was detected, and the spinal lesion status of the mice was detected by CT and HE (**Figure 1**).

### Preparation of hUCMSCs

hUCMSC was provided by Shanghai Quan Sheng Biotechnology Co., Ltd. The human umbilical cord was obtained from Dongguan Chang’an Hospital and passed ethical filing. The hUCMSCs were prepared by enzymatic digestion (Tryple, Gibco) from the umbilical cord of newborns and cultured in α-MEM medium containing 10% FBS, and after culturing to P6 generation, cell lyophilization solution was added according to the ratio of 1×10^8^ cells/10 ml and lyophilized by programmed cooling instrument. The preparation of hUCMSC is carried out in a sterile and clean GMP workshop.

### hUCMSCs cell phenotype and immunomodulatory function

Pre-life-storage hUCMSCs were taken for washing and counting. 3×10^6^ hUCMSCs were tested for surface markers including CD14, CD19, CD31, CD34, CD45, CD73, CD90, CD105, and HLA-DR. Surface markers of hUCMSCs were detected using flow cytometry (cytoFLEX, BeckmanCoulter).

To validate the immunomodulatory function of hUCMSCs, co-culture experiments were performed by incubating hUCMSCs with PBMCs extracted from different donors’ peripheral blood. The PBMCs were stimulated with PHA, and the inhibitory effect of hUCMSCs on PBMC proliferation, differentiation of Th cell subsets (Th1, Th17, T-reg), and secretion of inflammatory cytokines (IL-17A, TNF-α) were examined.

### hUCMSCs adipogenic, osteogenic and chondrogenic differentiation ability

MesenCult™ Lipogenic Differentiation Kit was used to detect the lipogenic differentiation ability of hUCMSCs. After 3 weeks of culture in lipogenic differentiation medium, cells were stained using Oil Red O stain and observed for lipid droplet formation under high magnification. The osteogenic differentiation ability of hUCMSCs was detected using MesenCult™Osteogenic Differentiation Kit. After 3 weeks of culture in osteogenic differentiation medium, cells were stained using Alizarin Red staining solution and observed under low magnification for the presence of visible red staining dots, and altered red staining dots for calcium nodules. The chondrogenic differentiation ability of hUCMSCs was measured using StemPro™ Chondrogenic Differentiation Kit. After 1 week of culture in chondrogenic differentiation medium, cells were stained using Alizarin Blue staining solution and observed for the presence of blue cartilage tissue.

### Th cells in mouse spleen

At the 20th week, the spleens of PGISp mice in each group were ground and prepared into single cell suspensions. After counting the cells, the cells were resuspended in 1640 medium (10% FBS) and seeded into cell culture dishes at a density of 5×10^6^ cells/ml. The next day, 1.5 µl of stimulation inhibitor was added to each sample, and cells were collected after 4-6 h of culture for flow cytometry (Celesta, BD). Th1, Th2, Th17 and T-reg detection indexes were CD3+CD4+IFN-γ+, CD3+CD4+IL4+, CD3+CD4+IL17A+ and CD3+CD4+CD25+FOXp3+, respectively. Among them, CD3, CD4, and CD25 are surface markers, IFN-γ and IL17A are intracellular factors, and FOXp3 is an intranuclear factor. The detection of intracellular factors and nuclear factors requires cells to be fixed and stained after membrane rupture.

### Thymus and spleen coefficient

The spleen and thymus of PGISp mice were collected at the 20th week of the experiment, and the organ weights were weighed using an analytical balance and divided by the corresponding body weight of each group of mice to calculate the spleen coefficient and thymus coefficient obtained.

### Inflammatory cytokines

Peripheral blood was collected from PGISp mice by eye blood sampling at week 20, and the serum was centrifuged at 10,000g for 15 minutes. The pro-inflammatory cytokines (INF-γ, TNF-α, IL-6, IL-17A, IL-22, IL-23, CCL-2) and anti-inflammatory cytokines (IL-10, TGF-β1) levels in peripheral blood were measured by the LEGENDplex Multifactor Assay Kit (Biolegend).

### Imaging score

The PGISp mice were anesthetized and examined by CT scan (Bruker micro-CT, Belgium) at the 13th and 20th weeks of the experiment, respectively, while the reference **Table 1** Bath Ankylosing Spondylitis Radiology Index (BASRI) Grade Classification and Scoring Criteria. The different mice were scored, with a score of 0 indicating no lesion, 1 indicating a slight lesion or not visible, 2 indicating the presence of tonic symptoms, and 3 indicating obvious tonic symptoms (38).

**Table 1:** BASRI grade classification and scoring criteria.

Score	Grade	Rating
0	Normal	No change
1	Suspicious or unclear	No clear change
2	Mild	On less than or equal to 2 vertebrae, with or without bony growth, any amount of erosion, squareness or sclerosis
3	Moderate	On greater than or equal to 3 vertebrae with osteophytes, with or without fusion of two vertebrae.
4	Serious	Fusion of greater than 3 vertebrae

### Pathological score

The lumbar vertebrae of mice were decalcified, paraffin-embedded, sectioned, HE stained, and sealed at week 20 after CT scan. The sections were browsed under the microscope or viewed digitally, and the tissue sections were carefully observed under 20x and 200x conditions, and the basic pathological changes in the sections such as congestion, bruising, hemorrhage, edema, degeneration, necrosis, hyperplasia, fibrosis, mechanization, granulation tissue, and inflammatory changes were described in text and reflected the differences between the pathological findings, while the typical lesion sites were arrowed with different colors to markings.

### Data analysis

Statistical analysis was performed with SPSS 22. The ROUT (Q = 1%) method was used to remove Outliers. Multigroup comparisons were analyzed using one-way ANOVA. A value of p < 0.05 was considered statistically significant. A value of p < 0.01 was considered statistically significant.

## Results

### PGISp mice

**Figure 2:**
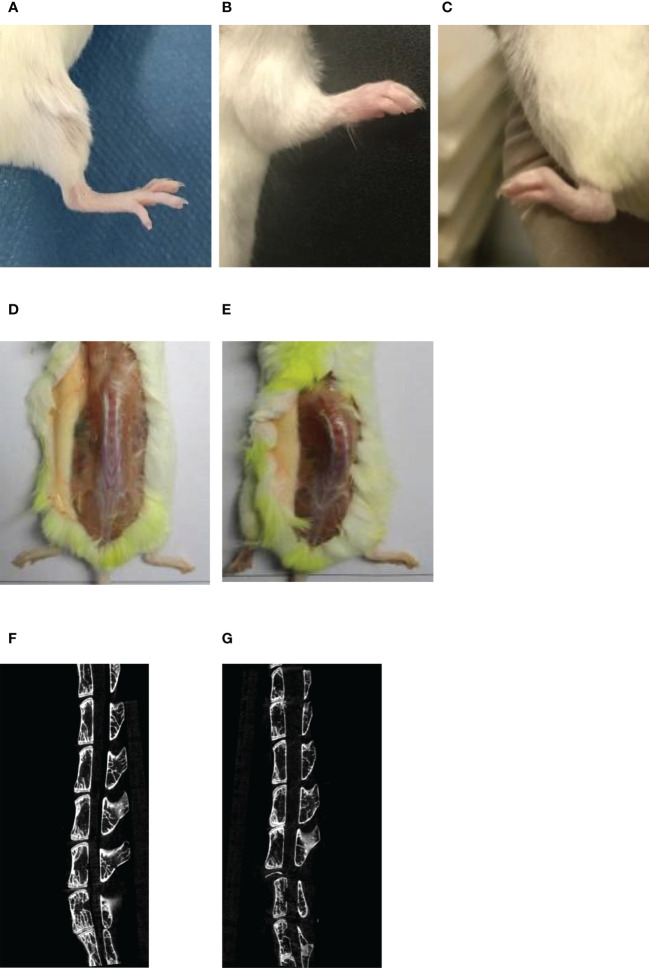
The symptoms of limbs and spines in normal/PGISp mice by taking pictures and CT scanning 13 weeks later, **(A)**: picture of normal hind limbs; **(B)**: picture of PGISp mice forelimb; **(C)**: picture of PGISp mice hind limbs; **(D, E)**: pictures of normal/PGISp mice spines, **(F, G)**: CT pictures of normal/PGISp mice spines.

60% of the mice induced by bovine proteoglycan combined with DDA started to develop peripheral joint disease and relieve symptoms at the 7th and 19th weeks, respectively, and about 40% of the mice showed no or very mild disease characteristics. At the 13th week, the limbs of the mice were observed, and the mice with no disease or mild disease were shaved. The limbs and spines of normal mice did not have any lesions (**Figures 2A, D**). PGISp mice showed thickened paws, redness and swelling of the limbs involved up to many joints and symptoms of lameness (**Figures 2B, C**), while the spines showed symptoms of dorsal arch and scoliosis (**Figure 2E**). Comparing the CT scan results of the normal group and the model group, the normal group had no symptoms of spinal lesions (**Figure 2F**), while the model group had blurred edges of the sacroiliac joints, sclerosis of the near-joint area, mild narrowing of the joint space, and symptoms of vegetation and joint fusion (**Figure 2G**).

### Purity, multiline differentiation and immunomodulatory function of hUCMSCs

**Figure 3:**
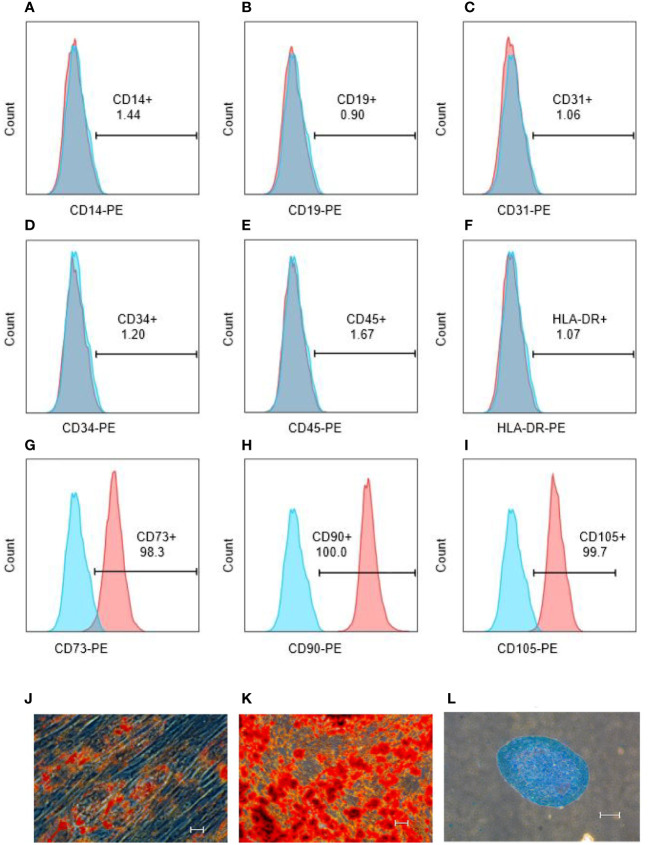
hUCMSCs surface markers detected by flow assay and three-lineage (adipogenic, osteogenic, chondrogenic) differentiation ability detected by immunocytochemistry before the injecting. **(A–F)**: the negative expression of surface markers CD14, CD19, CD31, CD34, CD45, HLA-DR of hUCMSCs; **(G–I)**: the positive expression of surface markers CD73, CD90, CD105 of hUCMSCs; **(J)**: Oil red O staining to analyze adipogenic differentiation of hUCMSCs; **(K)**: Alcian blue staining to explore chondrogenic differentiation of hUCMSCs; **(L)**: Alizarin red staining to analyze osteogenic differentiation of hUCMSCs. The scale bar = 50 μm in **(J)** and 200 μm in **(K, L)**.

Surface markers of hUCMSCs were detected by flow assay they showed that CD14, CD19, CD31, CD34, CD45, HLA-DR negative (**Figures 3A–F**) and CD73, CD90, CD105 positive (**Figures 3G–I**), and hUCMSCs were detected by immunocytochemistry for their lipogenic, osteogenic, and chondrogenic differentiation potential in the presence of different induction differentiation media. The results showed that hUCMSCs could also successfully transdifferentiate into adipocytes (analyzed by Oil Red O staining), osteoblasts (analyzed by Alizarin Red staining), and chondrocytes (analyzed by Arsenic Blue staining) (**Figures 3J–L**). The results indicate that hUCMSCs are characterized by high purity with multidirectional differentiation potential.

**Figure 4:**
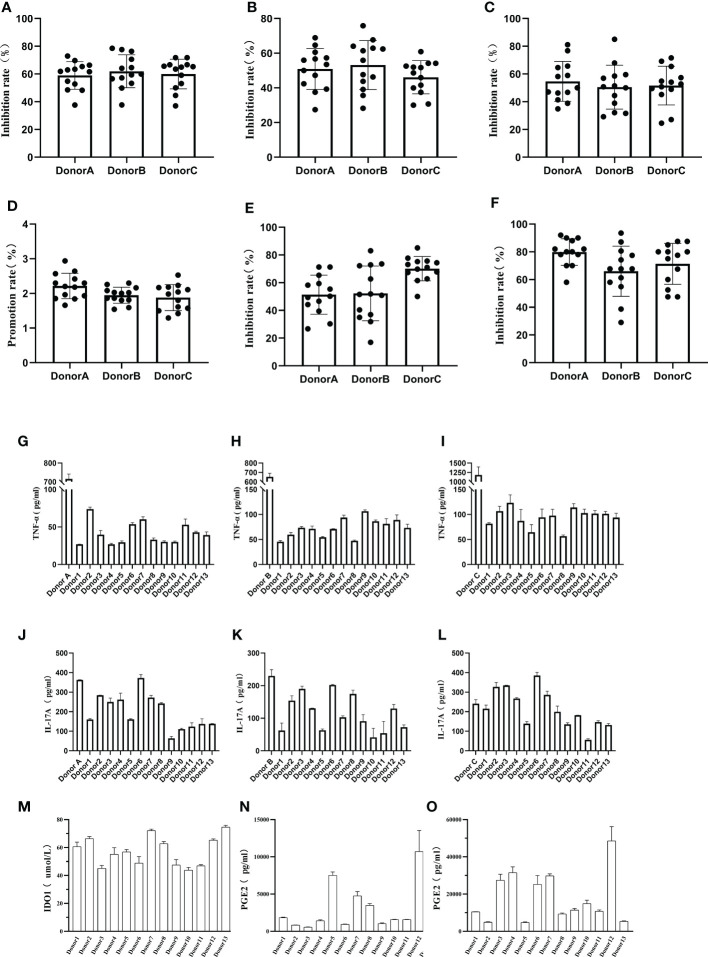
Detection of hUCMSCs immune regulation function. **(A–C)**: The effect of co-culturing hUCMSCs and PBMCs on the suppression function of PBMCs, CD3+ T cells, and CD4+ T cells; **(D–F)**: The effect of co-culturing MSCs and PBMCs on T-reg, Th1 and Th17 cells; **(G–I)**: Secretion levels of TNF-α after co-culturing hUCMSCs with PBMCs (Donor A, Donor B, Donor C), and the control group was PBMCs added with PHA; **(J–L)**: Secretion levels of IL-17A after co-culturing hUCMSCs with PBMCs (Donor A, Donor B, Donor C); **(M, N)**: The secretion levels of IDO1 and PGE2 in the culture medium after hUCMSCs were cultured for 24 hours; **(O)**: The secretion levels of PGE2 in the culture medium after hUCMSCs were cultured for 24 hours under the stimulation of TNF-α (500IU/ml) and IFN-γ (1000IU/ml). Proliferation rate = PBMC+PHA+MSC group/PBMC+PHA group-1; and the inhibition rate = (1-PBMC+PHA+MSC group/PBMC+PHA group) * 100%. PBMC : MSC =10:1, and 10ng/ml PHA was added.

Phytohaemagglutinin (PHA) has the function of promoting immune cell activation, by co-culturing hUCMSCs with PBMCs and stimulating with PHA, hUCMSCs demonstrated a proliferative inhibition effect on PBMCs, CD3+ T cells, and CD4+ T cells (**Figures 4A–C**). Additionally, they promoted T-reg proliferation and inhibited Th1 and Th17 proliferation (**Figures 4D–F**). Compared with the PBMC group stimulated by adding PHA, the secretion levels of TNF-α in the culture medium was significantly reduced under the co-culture condition of adding hUCMSCs (**Figures 4G–I**), and IL-17A also almost has a decreasing trend (**Figures 4J–L**). At the same time, we compared the ability of hUCMSCs prepared from umbilical cords from different donors to secrete IDO1 and PGE2 under the stimulation of inflammatory factors (TNF-α, IFN-γ). The results showed that hUCMSCs hardly secreted IDO1 without stimulation of inflammatory factors, but after stimulation with inflammatory factors, the secretion of IDO1 and PGE2 increased significantly (**Figures 4M–O**).

### Effects of hUCMSCs on T cell subsets in the spleen of PGISp mice

**Figure 5:**
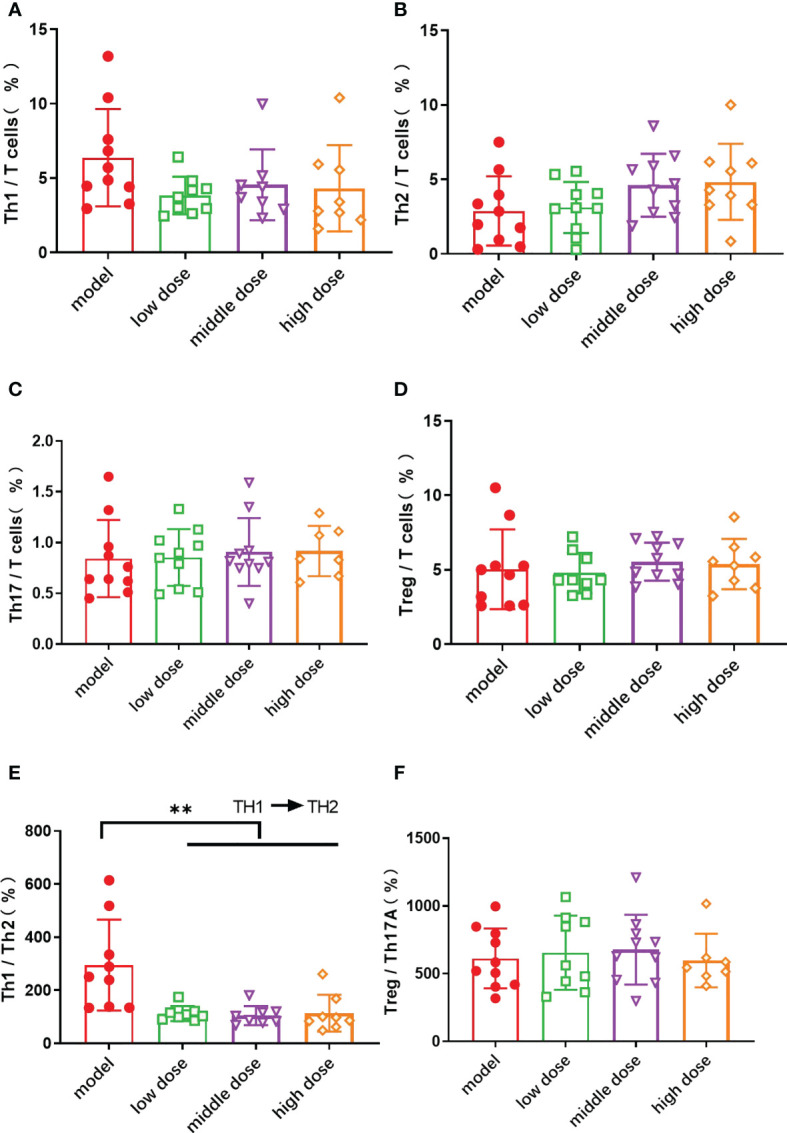
The proportion of Th cell subsets in CD3+ T cells in the spleen of mice in each group at the 20th week. **(A)**: the proportion of Th 1 cells in CD3+ T cells; **(B)**: the proportion of Th 2 cells in CD3+ T cells; **(C)**: the proportion of Th 17 cells in CD3+ T cells; **(D)**: the proportion of Treg cells in CD3+ T cells; Data are presented as mean ± SD. **p ≤ 0.01 vs. Model; **(E)**: the ratio of Th1 cells to Th2 cells; **(F)**: the ratio of Treg cells to Th17 cells. In model group, low dose group, middle dose group, n=10; in high dose group, n=9. Outliers were removed using the ROUT (Q = 1%) method, Th1: low dose (10.4), middle dose (17.7, 19.4), high dose (20); Th17: high dose (10, 2.78); Treg: low dose (17.8)、high dose (20);Th1/Th2%: model (1054.8)、low dose (981.1,816.7)、middle dose (600.6).

The spleens of mice in each group were collected after the 20th week, and each cell subpopulation in the spleen of mice was detected by flow assay. The results showed that the levels of Th1 cells was decreased in the treatment group as compared to the model group (**Figure 5A**), and the levels of Th2 cells was increased with no statistical significance (**Figure 5B**). The balance axis between Th1 and Th2 cells in the spleen was shifted towards Th2 cells after the injection of hUCMSCs (p<0.01) (**Figure 5E**). The proportion of Th17 cells and Treg cells in the spleen (**Figures 5C, D**) and the balance axis coefficient did not show differences (**Figure 5F**).

### Effects of hUCMSCs on thymus and spleen coefficients in PGISp mice

**Figure 6:**
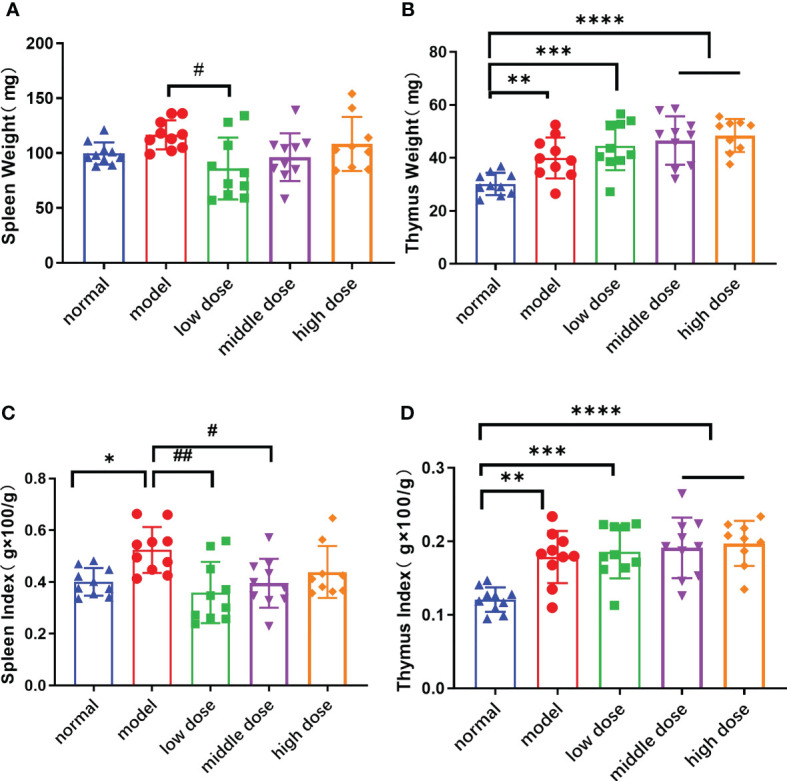
The weight and Index Score of Spleen/Thymus in each group at the 20th week. **(A)**: the spleen weight; **(B)**: the thymus weight; **(C)**: the spleen index, spleen weight (g)x100/mouse weight(g); **(D)**: the thymus weight(g) x100/mouse weight(g). Data are presented as mean ± SD; *p ≤ 0.05, **p ≤ 0.01, ***p ≤ 0.001, ****p ≤ 0.0001, vs. Normal; ^#^p ≤ 0.05, ^##^p ≤ 0.01, vs. Model; In model group, low dose group, middle dose group, n=10; in high dose group, n=9.

Compared with normal mice, the spleen and thymus weight (p<0.01) of PGISp mice showed an increasing trend. The thymus weight and thymus coefficient of the low-dose, middle-dose, and high-dose groups (p<0.001, p<0.0001, p<0.0001) also increased significantly (**Figures 6A, B, D**). The spleen of the mice in the low dose group was smaller than the model group (p<0.05). The spleen (p<0.05) and thymus coefficients (p<0.01) of PGISp mice were also significantly higher than those of normal mice. Compared with the model group, the mice in the low-dose group (p<0.01), the middle-dose group (p<0.05), and the high-dose group could inhibit the enlargement of the spleen, but had no significant effect on inhibiting the enlargement of the thymus (**Figures 6C, D**).

### Effects of hUCMSCs on the levels of blood inflammatory factors in PGISp mice

**Figure 7:**
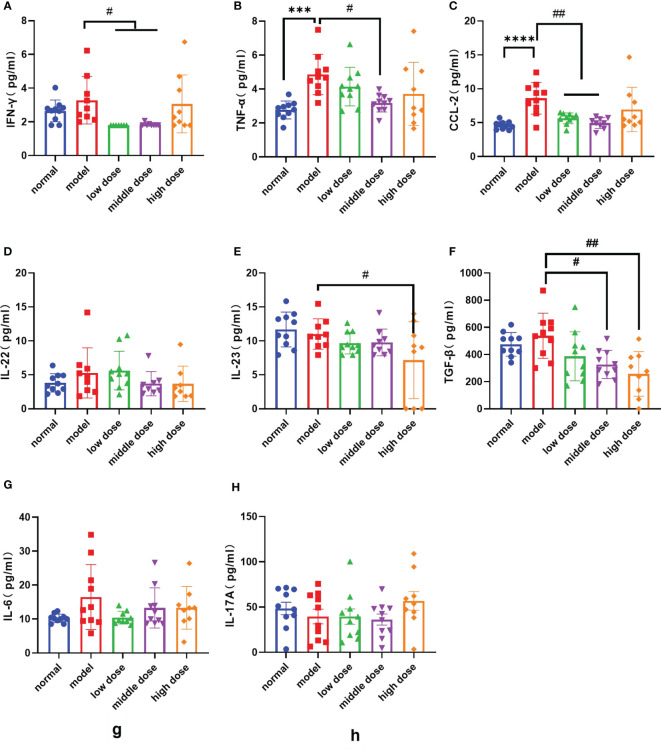
The levels of various factors in the serum of mice in each group at the 20th week, **(A)**: IFN-γ levels; **(B)**: TNF-α levels; **(C)**: CCL-2 levels; **(D)**: IL-22 levels; **(E)**: IL-23 levels; **(F)**: IL-17A levels; **(G)**: IL-6 levels; **(H)**: TGF-β levels. Data are presented as mean ± SD; ***p ≤ 0.001, ****p ≤ 0.0001 vs. Normal; ^#^p ≤ 0.05, ^##^p ≤ 0.01, vs. Model; In model group, low dose group, middle dose group, n=10; in high dose group, n=9. Outliers were removed using the ROUT (Q = 1%) method, IFN-γ: model (9), low dose (5.68, 4.32, 2.36), middle dose (2.28, 2.47); IL-22: model (23.85), middle dose (8.94), high dose (17.64); IL-23: model (24.38), middle dose (20.16); IL-6: low dose (32.46); IL: interleukin; TGF‐β1: transforming growth factor beta 1; CCL-2: The chemokine (C-C motif) ligand 2; IFN-γ: Interferon gamma.

Peripheral blood was collected from each group of mice after the 20th week, and the levels of pro-inflammatory factors and anti-inflammatory factors in the peripheral blood of mice were detected. Compared with the normal group, the PGISp mice expressed relatively high levels of TNF-α, CCL-2 (p< 0.0001, p< 0.0001), IL-6 and IL-22 in serum (**Figures 7B–D, G**), while the IFN-γ, IL-17A, IL-23 shows no statistically significant (**Figures 7A, E, H**). The IFN-γ levels in the serum of the low-dose group and the middle-dose group were lower than those of the model group (p< 0.05, p< 0.05), but the high-dose group showed no difference (**Figure 7A**); the TNF-α levels in the serum of each treatment group were lower than those of the model group, and the middle dose group had statistical significance (p<0.05) (**Figure 7B**); the CCL-2 levels in the serum of each treatment group were lower than the model group, and the low-dose group and the middle-dose group had statistical significance (p< 0.01, p< 0.01) (**Figure 7C**); the serum IL-22 levels in each treatment group showed a decreasing trend compared with the model group with no statistical significance (**Figure 7D**); the IL-23 levels in the serum of each treatment group showed a decreasing trend compared with the model group, and the high-dose group showed statistical significance (p< 0.05) (**Figure 7E**); the TGF-β levels in the serum of each treatment group showed a decreasing trend compared with the model group, and the middle-dose group and the high-dose group showed statistical significance (p< 0.05, p< 0.01) (**Figure 7F**); the levels of IL-6 and IL-17A showed no difference between the model group and the treatment group (**Figures 7G, H**).

### Effects of hUCMSCs on imaging scores in PGISp mice

**Figure 8:**
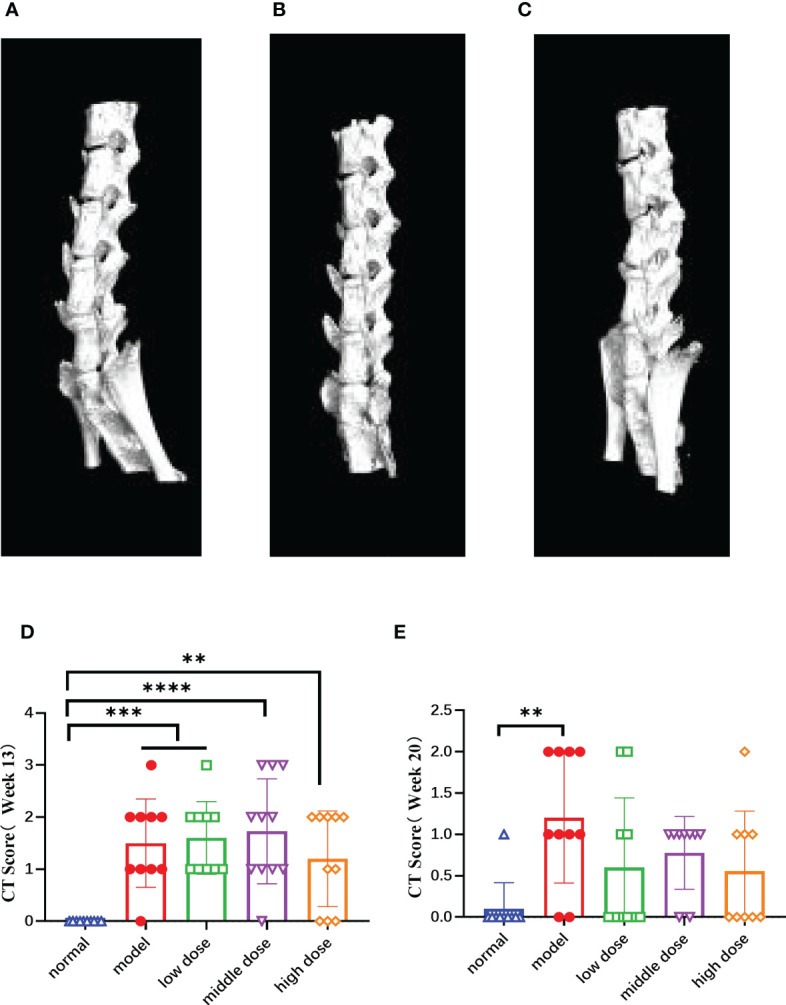
The CT images of the mouse spines in each group at the 20th week and score of lesion results referring to BASRI at the 13th week and the 20th week, **(A)** the CT pictures of the side of normal group; **(B)** the CT pictures of the side of model group, **(C)** the CT pictures of the side of hUCMSC treatment group, **(D, E)** the score of spinal lesions of the mice in each group at the 13th week and 20th week according to the BASRI grading scale. Data are presented as mean ± SD. **p ≤ 0.01 ***p ≤ 0.001 ****p ≤ 0.0001 vs. Normal. In model group, low dose group, middle dose group, n=10; in high dose group, n=9.

At week 20, frontal and lateral CT photographs were taken and the lesions were scored according to the BASRI scale. The spines of the normal group did not have any symptoms of pathological changes (**Figure 8A**). The model group showed heterotopic osteogenesis and significant fusion of the intervertebral tuberosities in (**Figure 8B**). hUCMSCs treatment groups initially inhibited the heterotopic osteogenesis activity and reduced the incidence of microarticular fusion in PGISp mice to a certain degree (**Figure 8C**).

After randomizing the model mice at the 13th week, we calculated the spinal pathology scores of the mice in each group according to the CT scan results (**Figure 8D**), and performed the same score at the same time at the 20th week (**Figure 8E**). The results showed that the model group, the low dose, the middle and the high group scores were significantly higher than those of the normal group (p< 0.001, p< 0.001, p< 0.0001, p< 0.01). The scores of mice in each group at the 13th week were significantly higher than those at the 20th week, showing the healing ability of the mice themselves. Compared with the normal group, the scores of the model group was still high (p< 0.01), while the treatment group have decreased tendency with no statistically significant.

### Effects of hUCMSCs on spinal pathology in PGISp mice

**Figure 9:**
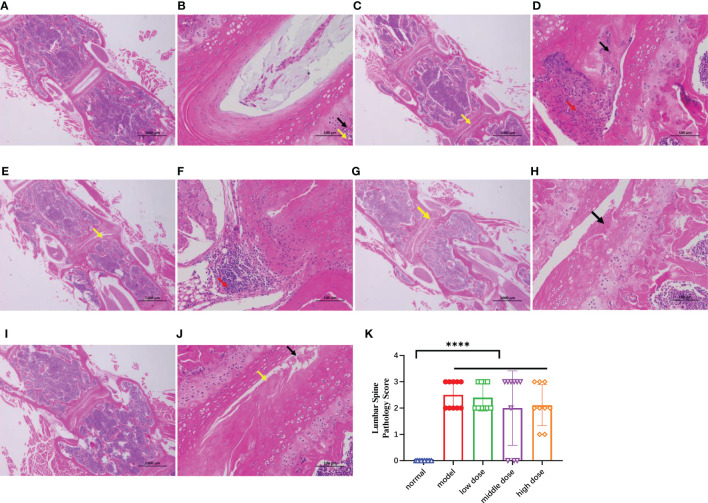
The HE staining results of the mouse spines in each group after paraffin embedding and longitudinal section at the 20th week, **(A, B)**: 20x and 200x photographed results of normal group, **(C, D)**: 20x and 200x photographing results of the model group, necrolysis of chondrocytes (black arrows) can be seen in multiple annulus fibrosus of the intervertebral disc, and cracks are formed (yellow arrows), with hyperplasia of connective tissue (red arrows) around; **(E, F)**: 20x and 200x photographing results of low-dose group, chondrocyte necrolysis (black arrows) can be seen in the annulus fibrosus of the intervertebral disc, and obvious Cleft formation (yellow arrows); focal infiltration of lymphocytes (red arrows) can be seen locally; **(G, H)**: 20x and 200x photographic results in the middle-dose group, necrolysis of chondrocytes in the annulus fibrosus of the intervertebral disc (black arrows), and obvious fissure formation (yellow arrow) can be seen. **(I, J)**: 20x and 200x photographing results in high-dose group, necrolysis of chondrocytes (black arrows) and obvious cleft formation (yellow arrows) can be seen in multiple annulus fibrosus of intervertebral discs; **(K)**: Mouse lumbar spines lesion score, Data are presented as mean ± SD. ****p ≤ 0.0001 vs. Normal. In model group, low dose group, middle dose group, n=10; in high dose group, n=9. .

After decalcification, paraffin embedding, sectioning, and HE staining of the lumbar vertebrae of mice by CT, the lumbar vertebrae of normal mice showed uniform tissue staining, intact disc structure, clear nucleus pulposus structure, regular fibrous ring arrangement, and normal chondrocyte morphology and structure under light microscopy (**Figures 9A, B**). In the model group, necrotic lysis of chondrocytes, formation of fissures, visible hemorrhage, surrounding connective tissue hyperplasia, and focal infiltration of lymphocytes were seen in the intervertebral disc fibrous rings of the diseased mice (**Figures 9C, D**). After treatment with different doses of hUCMSCs, the treatment group showed a certain tendency of symptom remission and less severe lesions than the model group (**Figures 9E–J**), and the results of lesion scoring showed that both the model group and the treatment group showed an increase in scoring compared with the normal group (p<0.0001). The middle dose and high-dose hUCMSCs have a certain function of relieving spinal lesions from the lesion scoring with no statistical difference (**Figure 9K**).

## Discussion

AS is a serum-negative spondyloarthropathy that is typically associated with chronic inflammation involving dendritic cells (DCs), macrophages, natural killer (NK) cells, and adaptive immune cells. These immune cells produce various innate cytokines that play a crucial role in the development of AS (39, 40). Recent studies have shown that there are significant autoimmune regulatory abnormalities in AS patients (41), and the decreased immunoregulatory function of bone marrow-derived mesenchymal stem cells (BMSCs) in AS patients is likely the initiating factor for its pathological occurrence. Compared to normal individuals, AS patients’ stem cells often exhibit enhanced pro-inflammatory ability and reduced anti-inflammatory function (42), and there is a dysregulation of cytokine secretion, such as increased levels of pro-inflammatory factors IL-17, IL-23, IL-22, and decreased levels of anti-inflammatory factors TGF-β, PD-L1, and IL-10 (11, 43). AS patients’ stem cells also have weakened ability to suppress macrophage activation, and there is an imbalance of peripheral blood Th17/Treg in AS patients, with a significant increase in Th17 cells and a decrease in Treg cells (12, 44). Additionally, AS patients’ BMSCs have enhanced osteogenic differentiation ability, and during their pathological osteogenesis process, they secrete more monocyte chemoattractant protein-1 (MCP-1), leading to dysfunction of monocytes and increased secretion of inflammatory factors such as TNF-α, which exacerbates the inflammatory environment in AS patients (45, 46). In summary, ankylosis is closely related to abnormal function of self-stem cells, and we boldly speculate that transplantation of exogenous stem cells may effectively improve the body’s inflammatory environment and repair abnormal states.

Stem cell therapy provides a feasible treatment option for AS patients who are unresponsive to traditional therapies. MSCs have significant tissue repair, tissue regeneration, and immune modulation properties. During stem cell therapy, stem cells can repair damaged joints through paracrine secretion of growth factors and regulate the immune system by upregulating Treg cell function, inhibiting T and B cell proliferation and differentiation, regulating NK cell activity, and blocking dendritic cell (DCs) maturation. *In vitro* experiments showed that co-culturing hUCMSCs with PBMCs under PHA stimulation exhibited the immune modulation function of hUCMSCs, suppressing T cell activation and proliferation, regulating Th cell subsets (Th1, Th17, T-reg), increasing secretion of anti-inflammatory factors (IL-10, IDO1, PGE2, and TGF-β) and decreasing secretion of inflammatory factors (TNF-a, IL-17A). In conclusion, we believe that stem cells may treat AS through the following mechanisms: 1) participate in the regeneration of damaged tissues and cells after intravenous infusion into patients; 2) have the ability to aggregate in the affected synovium, thereby promoting the repair of damaged joint tissues; 3) exhibit unique immune modulation functions by effectively inhibiting T lymphocyte proliferation and activation function through the addition of autologous and allogeneic MSCs to peripheral blood lymphocyte culture systems; 4) effectively inhibit the release of inflammatory mediators by exerting chemotaxis, thereby reducing inflammation in patients and improving clinical symptoms (8, 47, 48).

The safety and efficacy of mesenchymal stem cell therapy for autoimmune diseases, such as Systemic Lupus Erythematosus (SLE) and Multiple Sclerosis (MS), have been confirmed through multiple studies (49–51). Several clinical trials using mesenchymal stem cell transplantation to treat AS are currently underway to evaluate the safety and clinical effects of the transplantation in AS patients (8, 50). Current clinical trials and literature reports on stem cell therapy for AS indicate that MSC are safe and effective for the treatment of the disease.

Based on the above survey research, we conducted a preclinical study of hUCMSCs for the treatment of AS. We established a PGISp model by using a proteoglycan adjuvant. After three intraperitoneal injections of proteoglycan, some or all of the limbs and soles of the PGISp mice became thickened, red and swollen, and lameness, scoliosis, and arched back were formed. CT scanning and pathological examination showed blurred edges of spinal joints, sclerosis in the proximal joint area, mild narrowing of the joint space, and symptoms such as necrosis and dissolution of chondrocytes in the fibrous annulus of the intervertebral disc, and the formation of visible and obvious cracks. The blood levels of pro-inflammatory factors such as TNF-α and INF-γ were increased, as well as the spleen coefficient and thymus coefficient, and the proportion of Th1 cell subsets in the spleen was increased. However, 40% of the mice did not show or showed extremely mild symptoms. Mice treated with hUCMSCs had milder symptoms, less swelling of limb joints, milder spinal joint lesions such as lumbar spine and sacroiliac bone, lower levels of pro-inflammatory factors in blood, lower spleen coefficient, and decreased proportion of Th1 in the spleen, while the proportion of Th2 cells increased.

The thymus and spleen are important immune organs in the body, with the thymus being the central immune organ and the spleen being the peripheral immune organ. Changes in thymus and spleen indices are believed to reflect overall immune function, and abnormal increases in these indices are closely related to immune system overactivation. In the research and exploration of autoimmune diseases such as Rheumatoid arthritis (RA) and systemic lupus erythematosus (SLE) (52–54), immune organs such as the spleen, thymus, and lymph nodes have always been the focus of research. In this experiment, it was found that the immune organs of PGISp mice have significantly increased, and hUCMSCs treatment has a good function in inhibiting the spleen coefficient, but had no effect on the inhibition of the thymus coefficient. By detecting the Th cell subsets in the spleen, it was found that hUCMSCs treatment polarized the Th1 cells towards Th2 cells, but had no effect on Th17 and Treg cells. We speculate that this may be due to the short response time or low frequency of administration after hUCMSCs treatment. By detecting inflammatory factors in the blood, it was found that stem cells can effectively inhibit the inflammatory response in the blood, such as the decrease in levels of inflammatory factors such as CCL-2, TNF-a, and IFN-γ, but there was no difference in IL-17a and IL-22 inflammatory factor levels, which may be related to the sensitivity of the detection time points for these factors. We will focus on this aspect in future clinical studies.

To study the survival time and distribution position of hUCMSCs in mice, we conducted a tissue distribution study in normal mice using oxine labeling to track 89Zr-labeled human umbilical cord mesenchymal stem cells (hUCMSCs) after tail vein injection. Micro-PET/CT scans were performed at five time points: 1 hour, 24 hours, 72 hours, 168 hours, and 240 hours (**Supplementary Figures 1A–E**). The results showed that hUCMSCs were mainly distributed in the lungs and remained there for a long time. There were also small amounts of hUCMSCs distributed in the liver, heart, kidneys, and spleen, with low distribution in other tissues and organs such as the brain, joints, and muscles. The highest concentration of hUCMSCs in each organ was observed at 24 hours and decreased thereafter, with almost no presence at 240 hours. In patients with AS who are in an inflammatory state, stem cells mainly participate in systemic therapy through paracrine immunomodulation.

Currently, the pathogenesis of AS is not clear, but can be classified into several categories based on existing evidence, including genetic factors, immune system, microorganisms, and structural damage to the spine. Epidemiological studies have shown a significant relationship between this disease and genetic and environmental factors. The PGISp model mainly simulates the inflammatory environment in mice and is not related to genetic factors. In the future, we plan to use gene editing to produce a mutation-induced model, but this method has a high modeling difficulty and low success rate. In addition, we have overlooked the protein or transcriptome detection of PGISp mouse joints, and we will focus on researching and exploring this aspect later.

With the increase in the number of AS patients and the number of patients who are intolerant or unresponsive to immunosuppressive agents, current treatment methods are inadequate. This experiment mainly provides a reference for clinical trials of hUCMSCs in the treatment of AS, and this hUCMSCs has obtained the approval from the China Drug Evaluation (CDE) and is currently in the recruitment stage of clinical trials.

## Data availability statement

The raw data supporting the conclusions of this article will be made available by the authors, without undue reservation.

## Ethics statement

The studies involving human participants were reviewed and approved by Dongguan Changan Hospital Ethics Committee (2019507150102035). The patients/participants provided their written informed consent to participate in this study. The animal study was reviewed and approved by Shanghai Institute of Pharmaceutical Industry (SYXK(沪)2019-0027).

## Author contributions

NQ, HWW, and HW designed, directed and supervised the entire study. CJ, QLv, and QLiu performed most of the experiments; FX, ZW, and HPZ analyzed all results; DS and HYZ wrote the manuscript. All authors contributed to the article and approved the submitted version.
