# Supplementary material for: Evaluation of preclinical efficacy of human umbilical cord mesenchymal stem cells in ankylosing spondylitis
Source: Front Immunol. 2023 Mar 30;14:1153927. doi: 10.3389/fimmu.2023.1153927 (PMC10101200; doi:10.3389/fimmu.2023.1153927)
Supplement: Supplementary file 1 [file DataSheet_1.pdf]

Evaluation of Preclinical Efficacy of Human Umbilical Cord Mesenchymal Stem Cells in Ankylosing Spondylitis

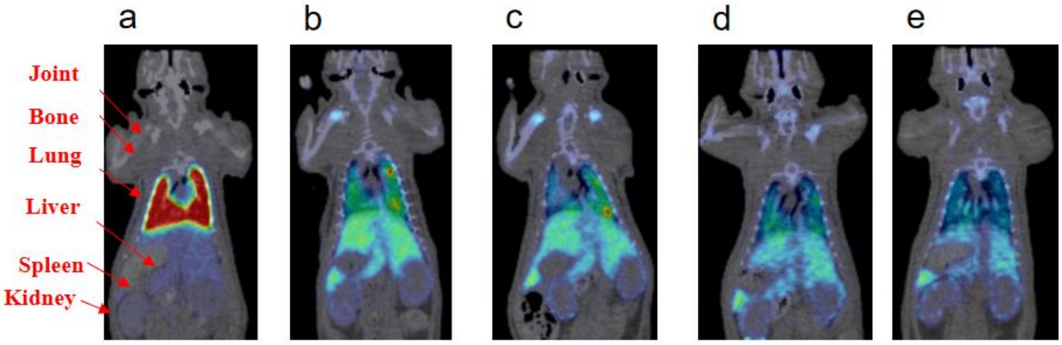

Supplementary Figure 1:PET/CT images of tissues/body fluids at different time points after a single tail vein administration of 89Zr-hUCMSCs. a-e: PET/CT results at 5 time points of 1 hour, 24 hours, 72 hours, 168 hours, and 240 hours.
